# A Noninvasive Score to Predict Liver Fibrosis in HBeAg-Positive Hepatitis B Patients with Normal or Minimally Elevated Alanine Aminotransferase Levels

**DOI:** 10.1155/2018/3924732

**Published:** 2018-10-14

**Authors:** Yanping Chen, Yanping Li, Na Li, Xiude Fan, Chunyan Li, Pingping Zhang, Qunying Han, Zhengwen Liu

**Affiliations:** ^1^Department of Infectious Diseases, First Affiliated Hospital of Xi'an Jiaotong University, Xi'an, 710061 Shaanxi, China; ^2^Department of Infectious Diseases, Affiliated Hospital of Yan'an University, Yan'an, 716000 Shaanxi, China

## Abstract

Noninvasive fibrosis tests are highly needed but have not been well studied in chronic hepatitis B patients with normal or minimally elevated alanine aminotransferase (ALT) levels. This study is aimed at developing a noninvasive score system to predict liver fibrosis in these patients. HBeAg-positive chronic hepatitis B patients with ALT levels of <80 IU/l and liver histology (*n* = 290) were assigned to training (*n* = 203) or validation (*n* = 87) groups. Training group patients were divided into nonsignificant (F0–1) and significant fibrosis (F2–4) according to METAVIR stages. Logistic regression was performed to identify factors for liver fibrosis and develop a score system. The capacity of the score to identify the severity of fibrosis was displayed by receiver operating characteristic curve (ROC) and area under ROC (AUROC) values. Multivariate logistic regression showed that HBeAg (ratios of the sample to the cutoff values (S/CO)) and liver stiffness measurement (LSM; kilopascals (kPa)) were independent factors of liver fibrosis. A score system composed of HBeAg and LSM by assigning a point of 1, 2, or 3 to different HBeAg and LSM levels, respectively, was developed. The scores 2-3, 4, and 5-6 of the sum of HBeAg and LSM points indicated nonsignificant, indeterminate, and significant fibrosis, respectively. The score system had an AUROC of 0.880 and showed similar performance in validation group patients. The accuracy for identifying significant and nonsignificant fibrosis was 77.14% in validation group patients and 71.26% in the entire group of patients. It is suggested that this noninvasive score system can accurately predict hepatic fibrosis and may reduce the need for liver biopsy in HBeAg-positive patients with normal or minimally elevated ALT levels.

## 1. Introduction

Chronic hepatitis B virus (HBV) infection may induce chronic hepatitis, liver cirrhosis, and hepatocellular carcinoma (HCC) and affects nearly 250 million people worldwide [1]. The course of chronic HBV infection is a dynamic process in which the individuals with HBV infection may experience different clinical phases displaying variable levels of serum alanine aminotransferase (ALT), HBV DNA, and HBV antigens [2]. The levels of serum ALT and HBV DNA and the severity of hepatic fibrosis are important predictors of long-term outcome and indicators of treatment initiation and response assessment [2, 3].

The indication of fibrosis or inflammation in patients with highly elevated ALT levels or cirrhosis is relatively easier. However, in HBV-infected patients with persistent borderline normal or slightly elevated ALT levels, liver biopsy is recommended [2] since it remains the gold standard for the evaluation of liver fibrosis and inflammation in chronic liver disease [4]. Liver biopsy is an invasive examination with high cost and certain pitfalls such as discomfort in patients, association of complications, and sampling errors [5, 6]. It is particularly difficult for patients to undergo multiple liver biopsies in order to serially monitor disease progression or to guide treatment. As a result, noninvasive approaches using readily available and noninvasive parameters have been developed for assessing the extent of liver fibrosis [7–13]. However, most of these methods were developed and used for hepatic fibrosis in hepatitis C virus (HCV) infection and/or HCV/human immunodeficiency virus (HIV) coinfection. Validation and comparisons of these models for liver fibrosis in chronic HBV infection showed unideal or inconsistent findings or diminished accuracy [14–22].

Recently, a model, gamma-glutamyltranspeptidase- (GGT-) to-platelet ratio (GPR), was developed to predict significant liver fibrosis and cirrhosis in patients with chronic HBV infection [23]. Some studies indicated that GPR had better performance in comparison with aspartate aminotransferase- (AST-) to-platelet ratio index (APRI) and fibrosis index based on 4 factors (FIB-4) in predicting liver fibrosis of chronic hepatitis B [24] and in improving the sensitivity and specificity of hepatic fibrosis assessment in chronic hepatitis B when combined with APRI or FIB-4 [25]. However, a study comparing seventeen noninvasive models including GPR showed that the models evaluated were not appropriate for all situations of chronic HBV infection [21]. Other studies showed that GPR does not add any advantage over APRI and FIB-4 in identifying significant fibrosis, severe fibrosis, and cirrhosis in chronic hepatitis B patients [26–28].

Few studies have been conducted to develop or evaluate noninvasive approaches in identifying liver fibrosis in hepatitis B e-antigen- (HBeAg-) positive chronic hepatitis B patients with normal or minimally elevated ALT levels (usually ALT of <2 upper limit of normal value (ULN)). APRI and FIB-4 were not indicated to be ideal hepatic fibrosis markers in these patients [20]. A model, globulin-platelet model (GP), was shown to be a more accurate noninvasive fibrosis model than APRI and FIB-4 to diagnose significant fibrosis and cirrhosis in chronic hepatitis B patients with high HBV DNA and mildly elevated ALT levels [29], but it has not been validated. Notably, liver stiffness measurement (LSM) by transient elastography, a noninvasive technique, was shown to be superior to APRI and FIB-4 for indicating liver fibrosis in HBV-infected patients with persistently normal ALT levels [20]. LSM was also shown to be a reliable noninvasive examination for the diagnosis of liver fibrosis in HBeAg-positive patients with high HBV DNA and normal or mildly elevated ALT levels [30]. However, the value of other noninvasive demographic, clinical, and laboratory parameters, especially their combination with LSM, needs to be further clarified in identifying liver fibrosis in HBeAg-positive patients with ALT of <2× ULN. Therefore, this study is aimed at developing a noninvasive approach by including LSM and routinely available noninvasive parameters in the analysis for the evaluation of liver fibrosis in HBeAg-positive patients with ALT of <2× ULN based on the fibrosis staging by liver biopsy. The results were also compared with the most discussed models, APRI and FIB-4, and the newly developed GPR.

## 2. Materials and Methods

### 2.1. Patients

The data of 290 HBeAg-positive chronic hepatitis B patients undergoing liver biopsy were collected from the Affiliated Hospital of Yan'an University from October 2013 to February 2018. Chronic HBV infection was defined as the persistent positivity of hepatitis B surface antigen (HBsAg), HBeAg, and antibody to hepatitis B core antigen (anti-HBc) for more than 6 months [31].

Inclusion criteria of patients were age ≥ 18 years; HBsAg, HBeAg, and HBV DNA positive for more than 6 months; serum ALT of <2× ULN; and signed informed consent for liver biopsy. Exclusion criteria of patients were: coinfection with other hepatotropic viruses such as HCV, hepatitis A virus, hepatitis E virus, or other viruses such as HIV; existence of other liver diseases including alcoholic, nonalcoholic, drug-induced, autoimmune liver diseases, and decompensated liver cirrhosis or tumors; diseases causing extrahepatic organ fibrosis; a history of antiviral therapy (interferon or nucleos(t)ides); pregnancy; and use of anticoagulant 1 week before liver biopsy. This study was performed to conform to the Declaration of Helsinki and was approved by the Ethics Committee of the Affiliated Hospital of Yan'an University (No. 2016–30).

### 2.2. Laboratory Tests

Fasting vein blood was collected for laboratory tests within 1 week of liver biopsy. Hyaluronic acid (HA), laminin, type III procollagen (PCIII), and type IV collagen (IV-C) were determined using Autodesk Chemiluminescent Immunoassay Analyzer (Yantai, China) and reagents from Beijing Yuande Bio-Medical Engineering Co., Ltd. (Beijing, China). HBV DNA quantification was performed using Abbott RealTime HBV assay with the Abbott m2000 SystemDNA reagents (Abbott Molecular Inc., Des Plaines, IL, USA) and ABI7500 Quantitative Cycler (ABI, USA). HBsAg was quantitatively determined by chemiluminescent microparticle immunoassay (CMIA) of ARCHITECT HBsAg assay (Abbott Ireland, Sligo, Ireland). HBeAg and anti-HBc were determined by CMIA of ARCHITECT HBeAg assay (Abbott GmbH & Co. KG, Wiesbaden, Germany) and anti-HBc II assay (Abbott GmbH & Co. KG, Wiesbaden, Germany), respectively, and the ratios of the sample to the cutoff values (S/CO) were used as HBeAg and anti-HBc levels, respectively.

### 2.3. Imaging Examination and Liver Stiffness Measurement

The thickness of the spleen and the inner diameter of the portal vein were measured using a Toshiba Ultrasound (Toshiba, Japan). LSM (kilopascals (kPa)) was performed using a liver transient elastography FibroScan System (Echosens SAS, Paris, France). The operator was blinded to the clinical characteristics of the patients. The median value of LSM was used to represent liver stiffness.

### 2.4. Liver Biopsy and Pathological Diagnosis

Percutaneous liver biopsy was performed with local anesthesia under the guidance of ultrasonography. Liver samples of about 1.5–3 cm in length were fixed with 10% formaldehyde solution and embedded with paraffin for histological analysis. Liver histology was interpreted by two hepatology pathologists who were blinded to the clinical information and the results of noninvasive tests. According to the METAVIR scoring system [32], liver fibrosis was classified into five stages: F0, no fibrosis; F1, portal fibrosis without septa; F2, portal fibrosis with rare septa; F3, numerous septa without cirrhosis; and F4, cirrhosis.

### 2.5. Grouping of Training and Validation Patients

The study population was divided into a training group and a validation group according to the recruiting time. Patients recruited from October 2013 to October 2016 were included in the training group (*n* = 203) and patients recruited from November 2016 to February 2018 (*n* = 87) were included in the validation group. According to the METAVIR scoring system [32], liver fibrosis was classified as nonsignificant (F0–1) and significant fibrosis (F2–4).

### 2.6. Statistical Analysis

SPSS version 20.0 software (SPSS Inc., Chicago, IL, USA) was used for statistical analysis. Data with normal distribution were expressed as the mean ± standard deviation (SD), and comparison was performed by *t*-test. Data with nonnormal distribution was expressed as median and interquartile range (median (IQR)). Comparisons were performed using a nonparametric test (Mann–Whitney *U* test). The dichotomous data were expressed by composition ratio. The comparison was carried out by *χ* ^2^ test. Liver pathological examination of fibrosis was used as the gold standard, and risk factors for liver fibrosis were analysed by logistic regression. A score system was derived from the independent factors. The diagnostic ability of the score system for liver fibrosis was evaluated using the receiver operating characteristic curve (ROC). The cutoff values were determined by the ROC at maximum Youden's index with optimal sensitivity and specificity. The area under the ROC (AUROC) was compared using MedCalc Software. A two-tailed value of *p* < 0.05 indicated a statistical significance.

## 3. Results

### 3.1. Patient Characteristics

Of the 290 HBeAg-positive chronic hepatitis B patients, 218 patients (75.2%) had nonsignificant hepatic fibrosis (F0–1) and 72 patients (24.8%) had significant hepatic fibrosis (F2–4) according to the liver pathological fibrosis stage.

The characteristics of patients in the training and validation groups and the differences between the two groups are shown in Table 1. Of note, the training group had a higher percent of patients with fibrosis F0–1 (80.79%, F0–1/F2–4 = 164/39) than the validation group (62.07%, F0–1/F2–4 = 54/33, *p* = 0.001, Table 1).

### 3.2. Noninvasive Parameters Associated with Hepatic Fibrosis in the Training Group

The fibrosis stages in the 203 training group patients were as follows: F0, 13 patients (6.4%); F1, 151 patients (74.4%); F2, 28 patients (13.8%); F3, 9 patients (4.4%); and F4, 2 patients (1.0%). Scilicet, 164 (80.8%) patients, had nonsignificant liver fibrosis (F0–1 group) and 39 patients (19.2%) had significant hepatic fibrosis (F2–4). The comparison of parameters between the patients with F0–1 and those with F2–4 fibrosis are shown in Supplementary Table 1. Compared with nonsignificant fibrosis patients (F0–1), patients with significant liver fibrosis (F2–4) had lower HBV DNA, HBsAg, HBeAg, and platelet count levels and higher anti-HBc, alpha-fetoprotein (AFP), AST, globulin, GGT, laminin, IV-C, LSM, and spleen thickness (Supplementary Table 1).

Multivariate logistic regression showed that lower HBeAg level (OR: 0.391, 95% CI: 0.241–0.632, *p* < 0.001) and higher LSM (OR: 1.522, 95% CI: 1.274–1.819, *p* < 0.001) were independent predictors of significant liver fibrosis (Table 2).

### 3.3. Development of a Score System and Performance in the Training Group

Based on multivariate logistic regression, a formula, *P* = *e* ^logistic^/(1 + *e* ^logistic^), was derived, where logistic = −1.988–0.94 Lg HBeAg + 0.42 LSM. By compound function derivation and operation [33, 34], the formula was optimized as follows: *P* = 1.6375–0.4326 HBeAg + 0.4451 LSM. Because the coefficients of the variables HBeAg and LSM were almost equal and their ratio approximated 1, the formula was simplified as *P* = −HBeAg + LSM to facilitate calculation.

The lower and upper cutoff values for HBeAg were 1247.38 S/CO and 106.91 S/CO, respectively, and the values for LSM were 4.95 kPa and 8.50 kPa, respectively (Table 3). To develop a score system, HBeAg and LSM were, respectively, assigned to different points from 1 to 3 according to the cutoff values, and the sum of HBeAg and LSM points (score) was used for staging liver fibrosis (Table 4). The scores 3 and 5 corresponding to a sensitivity of 92.3% and a specificity of 98.8% were determined to be the cutoff values of the score system, namely, scores 2–3, 4, and 5–6 indicated nonsignificant hepatic fibrosis, indeterminate fibrosis, and significant fibrosis, respectively (Table 4). The 203 patients in the training group were scored according to this system, and the AUROC was 0.880 (95% CI: 0.827, 0.921) for differentiating significant and nonsignificant fibrosis (*p* < 0.001, Supplementary Figure 1).

According to the pathological diagnosis of the liver tissue, the score for classifying hepatic fibrosis had a sensitivity of 90.0% and a specificity of 85.7%. The accuracy was 86.5%, positive predictive value was 58.7%, and negative predictive value was 97.4%. The positive likelihood ratio and negative likelihood ratio were 6.30 and 0.12, respectively.

**Figure 1:**
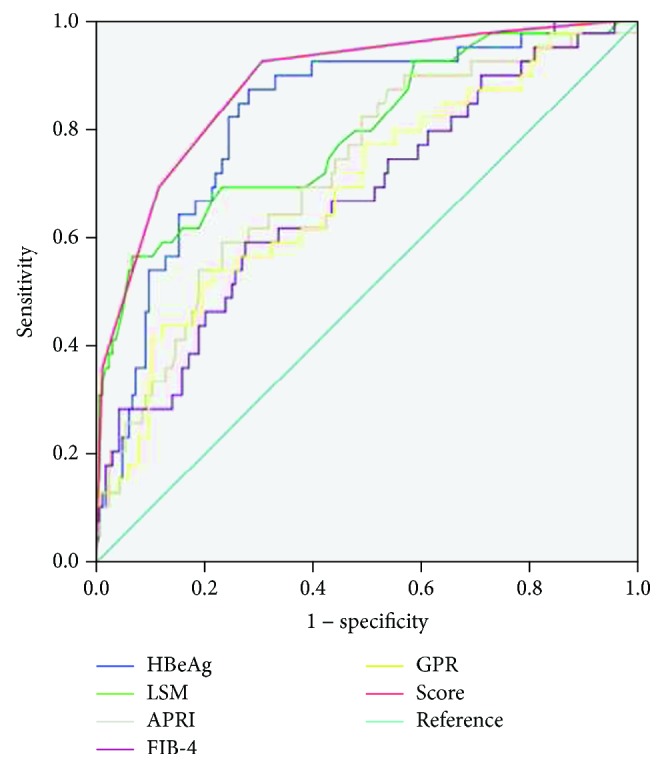
Comparison of the receiver operating characteristic curves of the score with HBeAg, LSM, aspartate aminotransferase- (AST-) to-platelet ratio index (APRI), fibrosis index based on 4 factors (FIB-4), and gamma-glutamyltranspeptidase-to-platelet ratio (GPR) for predicting significant from nonsignificant liver fibrosis in the training group patients.

The AUROC of the score (0.880) was significantly higher than HBeAg (0.822), LSM (0.791), APRI (0.720), FIB-4 (0.671), and GPR (*p* = 0.023, *p* = 0.0079, *p* = 0.0002, *p* < 0.0001, and *p* < 0.0001, respectively, Table 5, Figure 1).

### 3.4. Validation of the Score System

The fibrosis stages in the 87 validation group patients were as follows: F0, 11 patients (12.6%); F1, 43 patients (49.4%); F2, 23 patients (26.4%); F3, 9 patients (10.3%), and F4, 1 patient (1.1%), in which 54 patients (62.1%) had nonsignificant fibrosis (F0–1) and 33 patients (37.9%) had significant fibrosis (F2–4).

The AUROC of the score for predicting liver fibrosis in this group patients was 0.727 (0.612, 0.842). Because there were differences between the validation group and the training group patients in the fibrosis stages and the validation group had a relatively small sample size (Table 1), the AUROC was standardized by the methods as described elsewhere [35]. The adjusted AUROC was calculated to be 0.835 (95% CI: 0.612, 0.842), which had no significant difference compared with the training group (0.880, 95% CI: 0.827, 0.921; *z* = 0.677, *p* > 0.05, Supplementary Table 2).

Of the 87 patients in the validation group, 53 patients were predicted to have scores 2 and 3, 17 patients have score 4, and 17 patients have scores 5 and 6, respectively, according to the score system. Of the patients with scores 2–3, 41/53 (77.36%) had nonsignificant fibrosis and 13/17 (76.47%) patients with scores 5–6 had significant fibrosis. The total diagnostic accuracy of scores 2–3 and scores 5–6 was 77.14%. In the 17 patients with score 4, 9 cases had nonsignificant fibrosis and 8 cases had significant fibrosis. In the validation group, 62.07% of the patients can be accurately predicted for the degree of hepatic fibrosis by the score system.

## 4. Discussion

Previous studies showed that in HBeAg-positive patients with ALT of ≤2× ULN, the presence of significant liver fibrosis (≥F2) was 30.2% [36]. In HBeAg-positive patients with persistently normal ALT and ALT 1–2× ULN, significant fibrosis was found in 49.4% and 69.8% of the patients, respectively [37]. In HBeAg-positive patients with persistently normal or intermittently elevated ALT, histologic fibrosis stage of ≥2 was found in 40.2% and 65.5% of the patients, respectively [38]. In the present study, 24.8% (72/218) of the patients with ALT of <2× ULN had significant hepatic fibrosis (F2–4). Although there are differences in the proportion of liver fibrosis stages between the studies which may be related to the age and the definition of ALT levels, it is clearly revealed that nearly more than 25% of HBeAg-positive patients with ALT of <80 IU/l had significant liver fibrosis.

Many factors have been examined for the role in evaluating liver fibrosis in chronic hepatitis B patients. In our study, various demographic, clinical, and laboratory parameters were included in the analysis in relation to liver fibrosis in HBeAg-positive chronic hepatitis B patients with ALT of <2× ULN. Patients with significant liver fibrosis (F2–4), in comparison with nonsignificant fibrosis (F0–1), had lower HBV DNA, HBsAg, HBeAg, and platelet count levels and higher anti-HBc, AFP, AST, globulin, GGT, laminin, IV-C, LSM, and spleen thickness. These results are mostly consistent with previous studies showing that lower serum HBsAg, HBV DNA, and platelet count [39–42] and higher AFP, AST, globulin, GGT, laminin, and IV-C levels [29, 42–45] are associated with significant fibrosis in chronic hepatitis B patients. Older age was suggested to be related to significant fibrosis in chronic hepatitis B patients in previous studies [39, 41, 42, 46, 47]. However, our study did not show a relationship between patient age and liver fibrosis. This may be related to the patient population included in our study because HBeAg-positive chronic hepatitis B patients with ALT of <2× ULN are usually younger and the age range of the patients has small deviations. Higher ALT levels were also indicated to be associated with increased risk of advanced liver fibrosis in some previous studies [47, 48]. In the present study, ALT levels were not indicated to be a factor associated with liver fibrosis. The inclusion of patients with ALT of <2× ULN in the study may be partly related to this result because the magnitude of ALT fluctuation is small in this subgroup of patients. A previous study also showed that an elevated ALT level was not predictive of significant fibrosis for HBeAg-positive disease [41]. Moreover, the degree of liver fibrosis in chronic HBV infection is determined by complex interaction of multiple factors. A recent study showed that, in patients with ALT of >20 but ≤40 IU/l, age, ALT, and GGT were independent predictors of significant liver histological changes including significant fibrosis, while in patients with ALT of ≤20 IU/l, age was the only independent predictor of significant liver histological changes [46]. Albumin levels had no significant difference between patients with significant and nonsignificant fibrosis in HBeAg-positive chronic hepatitis B patients with ALT of <2× ULN in the present study. This is consistent with most of previous investigations. Anti-HBc and spleen thickness were rarely evaluated previously, and higher level of anti-HBc and wider thickness of spleen were observed in patients with significant hepatic fibrosis in the present study. Further studies are required to confirm the role of anti-HBc and spleen thickness in assessing liver fibrosis in chronic HBV infection.

Multiple logistic regression analysis using liver pathological examination of fibrosis as the gold standard showed that HBeAg levels and LSM are independent predictors of the hepatic fibrosis, and a novel score system composed of HBeAg and LSM levels was derived in the present study. HBeAg quantitation has been shown to have a moderate predictive value for discriminating immune tolerant phase and immune clearance phase in chronic HBV infection [49]. Serum HBeAg levels were indicated to be negatively correlated with the severity of liver inflammation in HBeAg-positive chronic hepatitis B patients [50], and high serum HBeAg levels were suggested to accurately predict insignificant histology among HBeAg-positive patients with ALT of <2× ULN [51]. HBeAg has been found to induce the activation and proliferation of hepatic stellate cells [52] which are pivotal players in the development of hepatic fibrosis. Consistently, HBeAg levels showed a good predictive value for liver fibrosis in HBeAg-positive patients with ALT of <2× ULN in the present study. It is suggested that HBeAg is involved in the pathology of liver fibrosis in chronic HBV infection and its levels may indicate the degree of liver fibrosis.

LSM by transient elastography is widely used for the noninvasive evaluation of liver fibrosis. It is indicated to be a reliable noninvasive test for the diagnosis of liver fibrosis in chronic hepatitis B patients with ALT of ≤2× ULN [30]. It is superior to current serobiomarkers in chronic hepatitis B patients with various levels of ALT [53, 54]. It is also superior to APRI and FIB-4 in chronic hepatitis B patients with persistently normal ALT levels [20]. Moreover, LSM is superior to FibroTest in the noninvasive identification of fibrosis among HCV carriers with normal/near-normal aminotransferases [55]. LSM displayed a good predictive value for liver fibrosis in HBeAg-positive patients with ALT of <2× ULN in the present study. Of note, enhanced inflammatory activity as indicated by elevated ALT can lead to elevated LSM values unrelated to histological fibrosis stage and can result in the overestimation of fibrosis [20, 53]. Therefore, LSM appears to have an advantage in classifying liver fibrosis in hepatitis B patients with ALT of <2× ULN because of the avoidance of potential influence by significantly elevated ALT levels.

The inclusion of parameters that directly reflect HBV replication (HBeAg) and reflect liver fibrosis (LSM) can better reflect the involvement of factors associated with liver fibrosis in HBeAg-positive patients with normal or slightly elevated ALT levels. In fact, the score system developed in this study is superior to the most used models APRI and FIB-4 and the newly developed GPR for predicting liver fibrosis in HBeAg-positive chronic hepatitis B patients with ALT of <2× ULN.

The score system was validated in the validation group patients with similar performance as in the training group patients. In addition to high sensitivity and specificity with high diagnostic accuracy and reproducibility, the score system is easy to calculate and simple to use and has better patient acceptance and higher speed of result obtainability with the noninvasive nature. This score may be especially useful for dynamic evaluation of liver fibrosis and may reduce the need for liver biopsy, making clinical care safer and more convenient for HBeAg-positive patients with ALT of <2× ULN.

Although the score system developed has advantages in evaluating liver fibrosis in this particular subgroup of patients, it is not perfect, in that the system has not been validated in large patient population with various demographic, clinical, and laboratory variations. For example, the patients included in our study had normal body mass index (BMI) while technical failure of LSM was shown to be common in patients with BMI ≥ 28 kg/m^2^ [56]. Therefore, additional studies evaluating the diagnostic accuracy of the score system are needed. In addition, the model may currently be unable to be adopted in some clinical practice settings, but it indicates an important direction toward more accurate evaluation of liver fibrosis with the increasing application of HBeAg determination and LSM usage.

In summary, the novel score system composed of noninvasive parameters, HBeAg and LSM, can accurately differentiate hepatic fibrosis and may reduce the need for liver biopsy in these subgroups of patients. Additional studies are needed to confirm the diagnostic accuracy of the score system and to evaluate the usefulness for identifying patients with significant fibrosis who might benefit from antiviral therapy.

## Figures and Tables

**Table 1:** Characteristics of patients in the training and validation groups.

	Training group	Validation group	*p*
*N*	203	87	
Age (years)	31 (26, 41)	34 (29, 40)	0.067
Gender (male/female)	121/82	50/37	0.735
BMI (kg/m^2^)	23.234 ± 3.337	23.550 ± 2.964	0.447
Family member of HBV infection (yes/no)	143/60	61/26	0.955
Smoking (yes/no)	41/162	24/63	0.167
Drinking (yes/no)	26/177	9/78	0.555
Lg HBV DNA (IU/ml)	7.544 (6.839, 7.968)	8.358 (6.340, 8.727)	<0.001
Lg HBsAg (IU/ml)	4.411 (3.811, 4.751)	4.424 (3.636, 4.834)	0.575
Lg HBeAg (S/CO)	3.098 (2.442, 3.162)	3.139 (2.217, 3.195)	0.103
Anti-HBc (S/CO)	9.800 (8.300, 11.000)	8.840 (7.440, 9.520)	<0.001
WBC (×10^9^/l)	5.700 (4.700, 6.510)	5.830 (4.920, 7.030)	0.219
RBC (×10^12^/l)	3.357 ± 1.246	4.904 ± 0.611	<0.001
Platelet (×10^9^/l)	198.515 ± 51.570	194.690 ± 58.548	0.579
AFP (ng/ml)	5.200 (3.400, 9.200)	2.670 (1.780, 4.140)	<0.001
ALT (IU/l)	32.000 (25.000, 46.000)	32.000 (21.000, 51.000)	0.661
AST (IU/l)	26.000 (20.000, 33.000)	28.000 (21.000, 35.000)	0.146
Tbil (*μ*mol/l)	11.000 (8.000, 15.000)	11.100 (7.900, 13.700)	0.526
Dbil (*μ*mol/l)	4.000 (3.000, 5.500)	4.400 (3.300, 5.800)	0.112
Albumin (g/l)	42.231 ± 4.617	40.872 ± 4.418	0.021
Globulin (g/l)	28.312 ± 5.293	28.339 ± 4.433	0.967
GGT (IU/l)	18.000 (12.000, 30.000)	18.000 (11.000, 25.000)	0.674
INR	1.052 ± 0.059	0.956 ± 0.060	<0.001
HA (ng/ml)	42.930 (17.000, 71.000)	50.290 (37.950, 62.220)	0.090
Laminin (ng/ml)	29.000 (8.000, 63.000)	50.040 (29.460, 72.130)	<0.001
IV-C (ng/ml)	34.000 (15.100, 58.000)	43.220 (23.420, 67.280)	0.014
PC-III (ng/ml)	2.000 (0.200, 4.000)	4.410 (3.320, 6.100)	<0.001
LSM (kPa)	5.400 (4.700, 6.500)	4.900 (4.200, 6.600)	0.063
Portal vein width (cm)	1.100 (1.110, 1.200)	1.200 (1.100, 1.200)	0.001
Spleen thickness (cm)	3.323 ± 0.593	3.393 ± 0.446	0.330
Liver fibrosis staging (F0–1/F2–4)	164/39	54/33	0.001

BMI: body mass index; WBC: white blood cell; RBC: red blood cell; AFP: alpha-fetoprotein; ALT: alanine aminotransferase; AST: aspartate aminotransferase; Tbil: total bilirubin; Dbil: direct bilirubin; GGT: gamma-glutamyltranspeptidase; INR: international normalized ratio; HA: hyaluronic acid; IV-C: type IV collagen; PC-III: type III procollagen; LSM: liver stiffness measurement.

**Table 2:** Results of logistic regression analysis of independent factors associated with liver fibrosis in the training group patients.

Variable	*B*	SE	Wals	Exp (*B*)	95% CI of exp (*B*)		*p*
					Lower	Upper	
Constant	−1.988	0.910	4.767	0.137	—	—	0.029
Lg HBeAg	−0.940	0.246	14.649	0.391	0.241	0.632	<0.001
LSM	0.420	0.091	21.417	1.522	1.274	1.819	<0.001

*B*: independent variable coefficient; SE: standard error; LSM: liver stiffness measurement; Lg HBeAg: HBeAg after log10 transformation.

**Table 3:** Cutoff values of HBeAg and LSM for classifying liver fibrosis.

Exploratory factor	Sensitivity (%)	Youden's index	Specificity (%)	Youden's index	Lower cutoff	Upper cut-off
HBeAg	92.3	0.527	90.2	0.440	1247.38^∗^	106.91^∗^
LSM	92.3	0.338	93.3	0.497	4.95^∗∗^	8.50^∗∗^

LSM: liver stiffness measurement. ^∗^Values in S/CO; ^∗∗^values in kPa.

**Table 4:** Point assignment of the score system for noninvasive diagnosis of liver fibrosis.

Exploratory factor	1 point	2 points	3 points
HBeAg (S/CO)	>1248	106–1248	<106
LSM (kPa)	<4.9	4.9–8.5	>8.5

LSM: liver stiffness measurement. Score = the point of HBeAg + the point of LSM. Score 2–3: nonsignificant fibrosis; score 4: indeterminate fibrosis; and score 5–6: significant fibrosis.

**Table 5:** Comparison of performance of the score system with HBeAg, LSM, APRI, FIB-4, and GPR for liver fibrosis.

	AUROC (95% CI)	SE	*Z*	*p* ^∗^
Score system	0.880 (0.827, 0.921)	0.0304		
HBeAg	0.822 (0.762, 0.872)	0.0365	2.273	0.023
LSM	0.791 (0.728, 0.845)	0.0443	2.654	0.0079
APRI	0.720 (0.653, 0.780)	0.0461	3.784	0.0002
FIB-4	0.671 (0.601, 0.735)	0.0502	4.247	<0.0001
GPR	0.687 (0.619,0.750)	0.0489	4.190	<0.0001

^∗^Compared with the score system. LSM: liver stiffness measurement; APRI: aspartate aminotransferase-to-platelet ratio index; FIB-4: fibrosis index based on 4 factors; GPR: gamma-glutamyltranspeptidase-to-platelet ratio.

## Data Availability

The data underlying the findings of this study are all included in the manuscript and in the Supplementary Materials section of this manuscript.

## Supplementary Materials

Supplementary 1 Table 1: comparison of patients with different liver fibrosis stages (F0–1 vs. F2–4) in the training group.

Supplementary 2 Table 2: comparison of AUC values of the score system for classifying liver fibrosis in training and validation group patients.

Supplementary 3 Figure 1: receiver operating characteristic curve of the score for identifying significant from insignificant liver fibrosis in the training group patients.
