# Supplementary material for: A Noninvasive Score to Predict Liver Fibrosis in HBeAg-Positive Hepatitis B Patients with Normal or Minimally Elevated Alanine Aminotransferase Levels
Source: Dis Markers. 2018 Oct 14;2018:3924732. doi: 10.1155/2018/3924732 (PMC6204156; doi:10.1155/2018/3924732)
Supplement: Supplementary 1 — Table 1: comparison of patients with different liver fibrosis stages (F0–1 vs. F2–4) in the training group. [file 3924732.f1.doc]

**Supplementary Table 1: Comparison of patients with different liver fibrosis stages (F0-1 *vs*. F2-4) in the training group**

|  | F0-1 | F2-4 | *p* |
| --- | --- | --- | --- |
| N | 164 | 39 |  |
| Age (years) | 31 (25, 41) | 34 (28, 42) | 0.316 |
| Gender (male/female) | 101/63 | 20/19 | 0.239 |
| BMI (kg/m^2^) | 23.142±3.251 | 23.626±3.694 | 0.417 |
| Family history of HBV infection (yes/no) | 116/48 | 27/12 | 0.854 |
| Smoking (yes/no) | 36/128 | 5/34 | 0.202 |
| Drinking (yes/no) | 23/141 | 3/36 | 0.288 |
| Lg HBV DNA (IU/ml) | 7.362±1.358 | 5.923±1.770 | < 0.001 |
| LgHBsAg (IU/ml) | 4.359±0.640 | 3.589±0.646 | < 0.001 |
| LgHBeAg (S/CO) | 3.118 (2.894, 3.175) | 1.991 (1.177, 2.856) | < 0.001 |
| Anti-HBc (S/CO) | 9.600 (8.085, 11.000) | 10.500 (9.200, 11.600) | 0.012 |
| WBC (×10^9^/L) | 5.725±1.542 | 5.984±1.955 | 0.443 |
| RBC (×10^12^/L) | 3.306±1.190 | 3.568±1.457 | 0.302 |
| Platelet (×10^9^/L) | 204.040±48.105 | 175.282±59.374 | 0.002 |
| AFP (ng/ml) | 4.850 (3.330,8.000) | 8.400 (5.000,12.000) | < 0.001 |
| ALT (IU/L) | 35.530±15.378 | 39.923±17.332 | 0.119 |
| AST (IU/L) | 26.683±9.347 | 32.692±11.393 | 0.001 |
| Tbil (μmol/L) | 11.000 (8.000, 14.625) | 11.000 (8.500, 16.000) | 0.639 |
| Dbil (μmol/L) | 4.000 (3.000, 5.300) | 4.000(3.000, 6.700) | 0.292 |
| Albumin (g/L) | 42.412±4.740 | 41.469±4.027 | 0.253 |
| Globulin (g/L) | 27.757±5.209 | 30.646±5.062 | 0.002 |
| GGT (U/L) | 17.000 (12.000, 27.750) | 26.000 (16.000, 37.000) | 0.006 |
| INR | 1.049±0.056 | 1.068±0.070 | 0.064 |
| HA (ng/ml) | 40.500 (15.450, 66.750) | 50.000 (22.000, 80.910) | 0.083 |
| Laminin (ng/ml) | 26.500 (5.000, 52.125) | 47.000 (15.000, 115.000) | 0.001 |
| IV-C (ng/ml) | 32.000 (13.500, 48.000) | 47.000 (23.000, 71.000) | 0.028 |
| PC-III (ng/ml) | 1.950 (0.200, 4.000) | 2.000 (0.200, 4.060) | 0.989 |
| LSM (kPa) | 5.200 (4.600, 6.100) | 8.800 (5.400, 11.900) | < 0.001 |
| Portal vein width (cm) | 1.100 (1.000, 1.200) | 1.100 (1.100, 1.200) | 0.119 |
| Spleen thickness (cm) | 3.255±0.590 | 3.555±0.567 | 0.004 |

WBC, white blood cell; RBC, red blood cell; AFP, alpha-fetaprotein; ALT, alanine aminotransferase; AST, aspartate aminotransferase; Tbil, total bilirubin; Dbil, direct bilirubin; GGT, γ-glutamyl transferase; INR, international normalized ratio; HA, hyaluronic acid; IV-C, type IV collagen; PC-III, type III procollagen; LSM, liver stiffness measurements.
