# Supplementary material for: A Noninvasive Score to Predict Liver Fibrosis in HBeAg-Positive Hepatitis B Patients with Normal or Minimally Elevated Alanine Aminotransferase Levels
Source: Dis Markers. 2018 Oct 14;2018:3924732. doi: 10.1155/2018/3924732 (PMC6204156; doi:10.1155/2018/3924732)
Supplement: Supplementary 2 — Table 2: comparison of AUC values of the score system for classifying liver fibrosis in training and validation group patients. [file 3924732.f2.doc]

**Supplementary Table 2: Comparison of AUROC values of the score system for classifying liver fibrosis in training and validation group patients**

|  | AUROC | SE | Z | *p* |
| --- | --- | --- | --- | --- |
| Training group | 0.880 (0.827, 0.921) | 0.032 |  |  |
| Validation group | 0.835^*^ (0.612, 0.842) | 0.059 | 0.677 | > 0.05 |

AUROC, area under the receiver operating characteristic curve; SE, standard error. * adjusted AUROC of the score.
