# Supplementary material for: A Noninvasive Score to Predict Liver Fibrosis in HBeAg-Positive Hepatitis B Patients with Normal or Minimally Elevated Alanine Aminotransferase Levels
Source: Dis Markers. 2018 Oct 14;2018:3924732. doi: 10.1155/2018/3924732 (PMC6204156; doi:10.1155/2018/3924732)
Supplement: Supplementary 3 — Figure 1: receiver operating characteristic curve of the score for identifying significant from insignificant liver fibrosis in the training group patients. [file 3924732.f3.pdf]

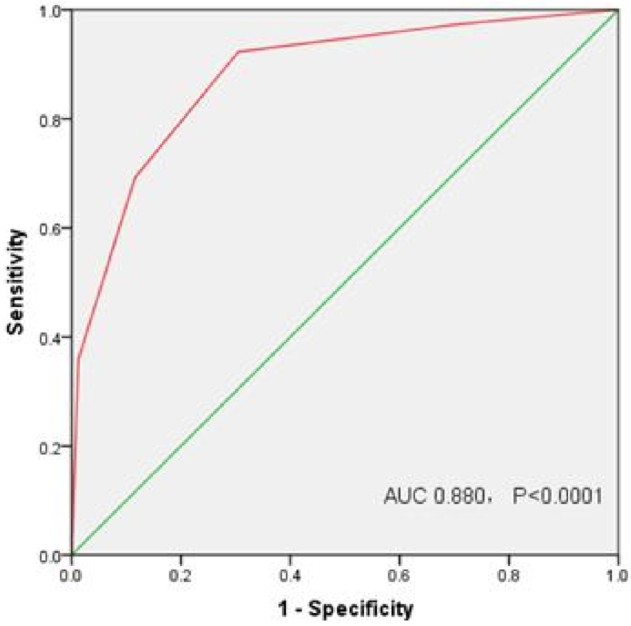

Supplementary Figure 1: Receiver operating characteristic curve of the score for identifying significant from insignificant liver fibrosis in the training group patients.
